# Physical Activity, Sedentary Behavior, and Satisfaction With Life of University Students in Qatar: Changes During Confinement Due to the COVID-19 Pandemic

**DOI:** 10.3389/fpsyg.2021.704562

**Published:** 2021-09-30

**Authors:** Souhail Hermassi, Lawrence D. Hayes, Ahmad Salman, Nilihan E. M. Sanal-Hayes, Emna Abassi, Lolwa Al-Kuwari, Nada Aldous, Nemah Musa, Amna Alyafei, El Ghali Bouhafs, René Schwesig

**Affiliations:** ^1^Department of Physical Education, College of Education, Qatar University, Doha, Qatar; ^2^School of Health and Life Sciences, University of the West of Scotland, Glasgow, United Kingdom; ^3^Department of Psychology, Lancaster University, Lancaster, United Kingdom; ^4^The Movement and Sport Research Center (CeRSM), University of Paris Nanterre, Nanterre, France; ^5^College of Arts and Sciences, Qatar University, Doha, Qatar; ^6^Department of Sports Science, Martin-Luther-University Halle-Wittenberg, Halle, Germany; ^7^Department of Orthopaedic and Trauma Surgery, Martin-Luther-University Halle-Wittenberg, Halle, Germany

**Keywords:** COVID-19, university student, public health, physical inactivity, sedentary behavior, home confinement, lifestyle and lockdown, life satisfaction

## Abstract

This study explored the effects of home confinement on physical activity (PA) and satisfaction with life (SL) among university students during the COVID-19 pandemic. A total of 531 subjects participated [male: *n*=203; female: *n*=328; age: 33.1±5.2years; mass: 72.1±17.5kg; height: 1.67±0.12m; and body mass index (BMI): 25.7±5.06 kg/m^2^]. Online survey questions considered “before” and “during” confinement. Confinement reduced all PA intensities (η_p_^2^=0.09–0.45, *p*<0.001) and increased daily sitting time (η_p_^2^=0.58, *p*<0.001). The largest reduction was in moderate intensity PA [metabolic equivalent of task-minutes/week (MET), η_p_^2^=0.45, *p*<0.001]. SQL decreased, with the score for “I am satisfied with my life” (η_p_^2^=0.42, *p*<0.001) decreasing from 28.4±5.7 to 20.6±9.7 arbitrary units (AU). Concerning SL, the largest change was detected for “the conditions of my life are excellent” (η_p_^2^=0.54, *p*<0.001). Time changes in all variables were demonstrative of large negative changes in both sexes. The difference in change between sexes was largest in terms of magnitude for the variable “the conditions of my life are excellent” (difference between groups, *Δd*=0.98). In sum, COVID-19 confinement reduced PA, heightened sitting time, and reduced SL in Qatar University students. This investigation could have a significant impact in developing PA guidelines for health maintainance during COVID-19 and successive pandemics in university students.

## Introduction

People are encouraged to adopt healthier lifestyles, and for this to become habitual, habits need to be implemented early in life for these to become rooted (Naudeau et al., 2008). Regular physical activity (PA) is one of the most effective ways of preventing premature death (Warburton et al., 2006; Warburton and Bredin, 2017). The World Health Organization (WHO) (2010) proposes a weekly minimum of 150min moderate exercise, 75min intense exercise, or a mixture of both (Guthold et al., 2018).

COVID-19 was first detected in China and has since spread throughout the world in a short period to affect millions of people, including nearly 4million deaths. However, the COVID-19 pandemic led to some populations being confined to their homes (Legido-Quigley et al., 2020). From March to April 2020, citizens of Qatar were banned from leaving the house for physical or social activities. During this time, this also meant other aspects of individuals’ lives were restrained due to the safety measures in place for controlling the spread of the virus. The Ministry of Education and Higher Education Qatar responded with robust emergency measures such as closing schools and using online platforms to virtually aid learning. Thus, ~30 million university students worldwide encountered a novel move from face to face traditional learning to virtual learning (Wang and Zhao, 2020; Zhang et al., 2021). The pandemic has brought not only the risk of death from viral infection but also public mental health problems, such as anxiety and depression symptoms worldwide (Wu et al., 2020).

With recent predictions indicating the COVID-19 pandemic could negatively impact people’s lifestyle with low levels of PA and increased sedentary behavior, with a resulting serious and detrimental effect on health if these behavioral manifestations persist (Bentlage et al., 2020). Trabelsi et al. (2021) reported that in quarantined individuals, time spent engaged in all PA and metabolic equivalents of tasks in each PA intensity zone decreased significantly during COVID-19 home confinement. In addition, the number of hours of daily sitting increased by ~2h/day during home confinement (Trabelsi et al., 2021). Furthermore, COVID-19-related home confinement significantly and deleteriously altered sleep quality and PA levels in a large global sample of people (Trabelsi et al., 2021). The study of Bastoni et al. (2021) highlights COVID-19 home confinement in Europe significantly impacted psychosocial wellbeing during the first wave of the pandemic, despite different lockdown intensities.

The review by Luzi and Radaelli (2020) suggests PA interventions have the potential to modulate inflammation, support the immune system, and improve vaccination outcomes. In the same way, another article summarized impacts of sedentarism on neuromuscular, cardiovascular, and metabolic health due to the COVID-19 home confinement. In addition, the work of Peçanha et al. (2020) described the social isolation and the increase in physical inactivity during the COVID-19 pandemic in the context of the global burden of cardiovascular disease. Taken together, these articles emphasize the importance of remaining physically active during the current pandemic and similar situations of extreme physiological and psychological stress.

However, no detailed study has examined the impact of university students’ change in PA, sedentary behavior, and SL during the COVID-19 pandemic. Thus, we believed it pertinent to investigate how adaptability (i.e., the capacity to adopt to changes, novel, and unpredictable situations accordingly) may impact commitment in PA and SL during the COVID-19 pandemic in Qatar. As universities integrated virtual learning to their curriculums, student engagement has suffered (Farooq et al., 2020; Nickerson and Shea, 2020; Perets et al., 2020). However, little research has examined factors that influence student PA engagement, QoL, and sedentary behavior in a pandemic.

Although the severe effect of the pandemic on students’ psychological well-being has been highlighted in previous studies, most of these are cross-sectional (Lau et al., 2005; Chen et al., 2020; Wu et al., 2020). To date, few longitudinal studies have explored decreased PA, SL, and symptoms of anxiety among university students during the COVID-19 pandemic (Wang and Zhao, 2020; Zhang et al., 2021). Documented changes in anxiety are divergent. For example, one study (Li et al., 2020) observed decreased symptoms of anxiety and depression after 2weeks of confinement, whereas two studies (Elmer et al., 2020; Zimmermann et al., 2020) indicated increased severity of anxiety. The authorized restrictions involving participation in outdoor physical activities such as regular exercise and PA during the COVID-19 outbreak have led to decreased PA and increased sedentary behavior, which in turn exacerbates common chronic health diseases, anxiety, and depression (Ammar et al., 2020a; Ingram et al., 2020; Hermassi et al., 2021a). Staying at home for prolonged periods can also increase sedentary behavior and decrease levels of PA and may lead to a mental health burden (Hemphill et al., 2020).

No previous studies have investigated PA levels and mental health of university students during the COVID-19 pandemic (Zhang et al., 2021), but one study has suggested 56% of Chinese students were involved in moderate to high levels of PA during the COVID-19 outbreak (Zhang et al., 2021). Qatari university students have attended virtual lectures and seminars, and their social lives were restricted due to enforced laws on remaining at home.

COVID19-related confinement is having a negative impact on mood-state, well-being, and lifestyle and eating behaviors (Ammar et al., 2020b,c). However, the negative changes in the majority of eating behaviors could be attributed to eating out of anxiety or boredom, a reduction in motivation to participate in PA or maintain healthy eating, or an increase in mood-driven eating (Ammar et al., 2021). Furthermore, physical inactivity is well recognized as a modifiable risk factor for several chronic diseases (Lee et al., 2012). A previous large-scale global investigation reported that physical inactivity causes 9% of premature mortality and 6–10% of the major non-communicable diseases (i.e., 6% of coronary heart disease, 7% of type 2 diabetes, 10% of breast, and 9% of colon cancers; Hermansen et al., 2019). In addition, previous studies have identified an increase in physical inactivity during the transition from adolescence to adulthood and throughout the college/university years (Crombie et al., 2009; Pullman et al., 2009; Kwan et al., 2012; López-Valenciano et al., 2021). Pengpid et al. (2015) estimated that prevalence of physical inactivity among university students in 23 low-, middle-, and high-income countries was 41%. Social distancing and confinements have largely altered the lifestyle of university students, and it is not clear how changes in the aforementioned factors are affecting PA levels of this population. In light of the above, more research is needed to clarify the effects of home confinement on PA, SL, and sedentary behavior. Previous studies have found a direct relationship between psychological distress, poor academic performance, and career outcomes (Toufexis et al., 2006; Raskind et al., 2019). Furthermore, anxiety symptoms can serve as building blocks for further detrimental mental health outcomes and diminished quality of life (Fichter et al., 2010; Kasteenpohja et al., 2018). As such, the primary aim of the present study was to examine the effects of home confinement on PA, sedentary behavior, and SL of Qatar university students during the COVID-19 outbreak. We hypothesized *a priori* that COVID-19 confinement would, independent of gender, (a) negatively impact PA participation of university students, (b) increased the sedentary behavior, and (c) negatively impact SL. Additionally, we expected levels of PA would be positively associated with SL.

## Materials and Methods

An important criterion for selecting questionnaires (e.g., PA and SL) is the evidence of validity and reliability (Diener et al., 1985; Craig et al., 2003; Lee et al., 2011). In this cross-sectional research, we used two questionnaires: the short version of the (1) “International Physical Activity Questionnaire” (IPAQ; Craig et al., 2003; Lee et al., 2011) and the (2) “Satisfaction with Life (SL; Diener et al., 1985). The Arabic and English versions of the questionnaires are publicly available. The purpose of the IPAQ is to enable validated and standardized estimations of physical activity. In our study, we will use the short version of IPAQ, which is open access, and no permissions are required to use it (Craig et al., 2003). The SL questionnaire is psychometrically sound with high internal consistency and temporal stability (Diener et al., 1985).

### Participants

This was an observational, cross-sectional study on public health. The study was completed in accordance with the Declaration of Helsinki. The Qatar University’s institutional review board (QU-IRB 1350-EA/20) approved the present study’s protocol. This study was carried out within the context of an intended larger study we were undertaking concerning lockdown on PA, sedentary behavior, and SL in Qatar. However, due to the state of alarm and dynamic characteristics of lockdown, recruitment of subjects was temporarily suspended, and we decided to report on the impact of lockdown on the population already recruited to increase internal validity. Target participants comprised of undergraduate and postgraduate students from the College of Business and Economics (CBE), College of Education (CED), College of Arts and Sciences (CAS), College of Health Sciences (CHS), College of Law (LAWC), and College of Engineering (CENG) of Qatar University students who were staying at home and engaged in online learning during the COVID-19 outbreak. We conducted a cross-sectional anonymous web-based survey to collect data from September 2020 to October 2020. From July 2020 to October 2020, the government of Qatar entered its second stage of the normalization plan and adopted a flexible lockdown.

Government guidelines in Qatar were altered as a response to the pandemic in early April, and citizens were prohibited to leave their homes to visit coastal areas, children’s playgrounds, parks, sport areas, recreation areas, and squares. Moreover, seeing more than two people was prohibited. The government prohibited citizens to leave their homes and to be on the streets without documents evidencing the purpose. Police was entrusted to overlook and monitor citizens and ensure citizens followed self-isolation regulations. Breaking these regulations was penalized with a fine or imprisonment. Educational institutions were solely based online. Face masks and social distancing measures were in place.

We gathered 531 responses in this cross-sectional study using an online survey that consisted of the IPAQ Short Form (IPAQ-SF) and SL Questionnaire. Both surveys were administered in English and Arabic. The survey consisted of 22 questions on demographic information (e.g., age, gender, body mass, and height), athletic status (e.g., defined as a person who competes in one or more activities that involve physical strength, speed, and/or endurance), smoking status, health status (e.g., anxiety or depression, diabetes, cardiovascular disease and pulmonary disease, and motor problem), PA (e.g., vigorous, moderate, and walking activity), and SL (e.g., life conditions, satisfaction with life, and important things for life). Questions were to be answered directly in sequence regarding “before” and “during” confinement conditions (Ammar et al., 2020a; Ingram et al., 2020). Measures were obtained on the same day to limit order or recall bias, considering the regularly evolving situation of the pandemic. Once the deadline for finalizing the survey passed, conflicting responses (inconsistency between data) and duplicated responses (≥ 2 submissions with equivalent responses in a short period) were removed from the database.

### Sample Size

The sample size was calculated according to the following predictive equation (Whitley and Ball, 2002).


(1) N = ((Zα/2 2 p q))/Δ2

N: number of needed participants,

*Z*α/2: two-tailed normal deviate for type 1 error,

*p*: change in % from “before” to “during” confinement,

*q*: equal to “1−p” and Δ: accuracy; where “*n*” was the number of needed participants,

“*Z*α/2” was the two-tailed normal deviate for type 1 error (*Z*α/2=1.96 for 95% level of significance),

“q” was equal to “1−*p*”,

“Δ” was the accuracy (=3%), and

“*p*” was the percentage of change in social participation from “before” to “during” confinement period.

Comparable to Ammar et al. (2020a), the “*p*” was chosen from a study (Zhang et al., 2021). Zhang et al. (2021) examined the immediate effects of the COVID-19 pandemic on mental health and quality of life. Based on these findings, it appeared that 57.8% (*p*=0.578) of subjects experienced an increase in shared feelings with family members (Zhang et al., 2021). Consequently, the calculated sample size for our investigation was *n*=540. Based on comparable studies with participation rates from 92% (Hermassi et al., 2021a) and 91% (Hermassi et al., 2021b), we assumed a dropout rate of 10% (*n*=54). Therefore, we aimed to recruit 600 participants.

### Experimental Design: Survey Development, Promotion, and Distribution

A steering group of academics and scientists (in the fields of sport science, human sciences, statistics, and computer science) designed the electronic survey at the University of Qatar (where the principal investigator was based). The questions of the survey were subsequently validated and amended by >30 scientific experts and colleagues. Survey was uploaded to Google™ platform and shared *via* same platform. The society shared the electronic survey uniform resource locator (URL) through different medias: electronic e-mails, shared on ResearchGate™, society’s faculties official pages, and LinkedIn™, and other social media platforms, such as Facebook™, WhatsApp™, and Twitter™.

The university students assisted in the dissemination of the survey through promoting the survey within their social platforms. In total, initially 600 participants received the URL of the online survey and of these 531 completed the survey and were included in the final analysis (participation rate: 92%). The survey consisted of an introductory page describing the aims and the background of the survey, an ethics page and a consent form, and the society and participants were given the option to complete the survey in either English or Arabic. Inclusion criteria included participants were aged ≥18years and in good physical health (no pain, diagnosis, and injury at time of examination). Participants were excluded in case of a positive COVID-19 test or cognitive deterioration.

### Data Privacy/Security

Participants were informed that data would be stored and used for solely research purposes. Responses of participants were kept anonymous and were confidential according to Google’s privacy policy.^1^ Participants were not required to provide names or contact information to ensure anonymity. Participants were free to withdraw and leave the questionnaire at any time before the final submission of responses. If they did so, responses would not be saved. Responses were saved once the “submit” button was pressed.

Upon acceptance of the survey objectives in the consent form section, participants preceded by clicking the “Next” button to start the survey or to withdraw their participation in the survey. This web-based survey was non-commercial and on voluntary bases.

By completing the survey, participants acknowledged the approval form and consented to voluntarily participate in this anonymous study. Participants were instructed to be honest with their responses.

### International Physical Activity Questionnaire Short Form

According to the official IPAQ-SF recommendations, data are summed within each item (i.e., vigorous intensity, moderate intensity, and walking) to estimate the total amount of time engaged in PA per week (Craig et al., 2003; Lee et al., 2011). Weekly PA (MET-min·week^−1^) was calculated by summing products of each PA item by a MET value specific to each category of PA. We assigned two sets of MET values. The first was the original values based on official IPAQ procedures for young and middle-aged adults (18–65years old): vigorous PA=8.0 METs, moderate PA=4.0 METs, and walking=3.3 METs. Additionally, we added the total PA (sum of performed vigorous, moderate, and walking activity) as a fourth item and sitting time as the fifth item of sedentary behavior assessed using the question: “Since self-isolating, how much time you have spent sitting daily?”

### Satisfaction With Life Questionnaire

The SL questionnaire is a crisis-oriented questionnaire to assess satisfaction with the respondent’s life before and during the confinement period. The SL questionnaire is based on a five-item scale designed to measure global cognitive judgments of one’s life satisfaction (Kasteenpohja et al., 2018). Participants indicate how much they agree or disagree with each of the five items using a seven-point scale that ranges from 7 strongly agree to 1 strongly disagree (Strongly agree=7; Agree=6; Slightly agree=5; Neither agree nor disagree=4; Slightly disagree=3; Disagree=2; and Strongly disagree=1). Though scoring should be kept continuous (sum of scores on each item), there are some cut-offs to be used as benchmarks (Kasteenpohja et al., 2018). The total score of this questionnaire is comprised of the sum of scores from individual items. The total score for the SL questionnaire ranges from 5 to 35, with lower scores corresponding to extreme dissatisfaction and higher scores with extreme satisfaction (Kasteenpohja et al., 2018).

### Statistical Analyses

SPSS version 25.0 for Windows was used to perform statistical analyses (SPSS Inc., IBM, Armonk, NY, United States). For the purpose of investigating gender differences for dichotomously or ordinally scaled data, a Chi-square test was used. Prior to analysis, all variables were tested for normality (Shapiro–Wilk test). To investigate differences in sexes and differences between times (before vs. during confinement), a two factor (sex, time) univariate general linear model was utilized. Greenhouse–Geisser correction (where sphericity was violated; Bortz, 1999) was used to calculate the ANOVA *p* values and partial eta-squared (η_p_^2^). Main effects for sexes, time (before, during confinement), and interaction (gender×time) effects were reported accordingly.

The effect size (the mean difference between scores divided by the pooled SD) was also calculated for each parameter (Hartmann et al., 1992). A positive effect size means an improvement of a variable, and a negative value indicates deterioration. Percentage changes were calculated as [(before value − during value)/before value]×100. The interpretation of effect sizes is based on Cohen’s thresholds for small effects (*d*<0.5), moderate effects (*d*≥0.5), and large effects (*d*>0.8; Cohen, 1988).

Differences between means (time effect) were considered as being meaningful, if *p*<0.001, η_p_^2^>0.10, and the effect size (d) was ≥0.8 (Richardson, 2011). Based on the number of parameters/tests and after applying a Bonferroni correction, we adjusted the α error level for both types of parameters (PA: 0.05/13=0.004; satisfaction of life: 0.05/6=0.008). In the interest of a uniform approach and conservative assessment of the effects, we used an alpha error level of *p*<0.001.

Pearson’s product moment correlations were calculated to evaluate the relationship between PA parameters and SQL parameters. Magnitude of correlation (*r*) between measures was interpreted as follows: <0.1=trivial; 0.1–0.3=small; 0.3–05=moderate; 0.5–0.7=large; 0.7–0.9=very large; and 0.9–1.0=almost perfect (Cohen, 1988). Data are reported as mean±SD.

## Results

### Normal Distribution

All variables were not normally distributed (*p*<0.001), and thus, Greenhouse–Geisser correction was applied to all ANOVAs.

### Sample Description

A total of 531 (female: *n*=328; male: *n*=203) participants were recruited in Qatar (Table 1).

**Table 1:** Demographic and anthropometric characteristics of all participants (*n*=531).

	Total (*n*=531)	Male (*n*=203)	Female (*n*=328)	Between gender	
				*p*	η_p_^2^
Age (years)	23.5±5.2 (17.0–65.0)	24.3±5.0 (17.0–48.0)	23.1±5.2 (17.0–65.0)	0.008	0.01
Height (m)	1.67±0.12 (1.09–1.98)	1.78±0.09 (1.45–1.98)	1.61±0.08 (1.09–1.89)	<0.001	**0.48**
Weight (kg)	72.1±17.5 (28.0–140.0)	84.5±15.2 (43.0–135.0)	64.4±14.1 (28.0–140.0)	<0.001	**0.31**
BMI (kg/m^2^)	25.7±5.1 (11.7–44.7)	26.8±4.8 (12.2–42.2)	25.0±5.1 (11.7–44.7)	<0.001	0.03

Meaningful effects (criteria: *p*<0.05, η_p_^2^>0.10, and *d*>0.8) are bolded.

Significant differences were observed between males and females for height and weight but not for age. Body mass index (BMI) was the anthropometric parameter with the smallest difference between males and females. The proportion of physically active among women was higher than among men, while the number of smokers was greater among men (Table 2). However, the anxiety or depression was the most prevalent health problems reported.

**Table 2:** Description of the sample (total: *n*=531; female: *n*=328, male: *n*=203) regarding demography, anthropometry, and health status depending on gender.

Variables	Categories	Male *n* (%)	Female *n* (%)	Total *n*	Chi^2^ (*p*)
Age (years)	18–35	197 (39)	315 (61)	512	0.815 0.665
	36–55	6 (33)	12 (67)	18	
	>55	0 (0)	1 (100)	1	
BMI (kg/m^2^)	<18.5	3 (15)	17 (85)	20	19.05 <0.001
	18.5–24.9	78 (31)	173 (69)	251	
	25–29.9	79 (45)	96 (55)	175	
	30 or greater	43 (51)	42 (49)	85	
Athlete	Yes	53 (30)	125 (70)	178	8.105 0.004
	No	150 (43)	203 (57)	353	
Smoker	Yes	47 (100)	0 (0)	47	83.315<0.001
	No	156 (32)	328 (68)	484	
Health status	None of the above	148 (38)	243 (62)	391	29.96<0.001
	Anxiety or depression	40 (60)	27 (40)	67	
	Diabetes	8 (35)	15 (65)	23	
	Cardiovascular disease and pulmonary disease	1 (5)	20 (95)	21	
	Motor problem	6 (21)	23 (79)	29	

### International Physical Activity Questionnaire Short Form

Time effects for PA parameters (vigorous or moderate physical activities and walking) and sedentary behavior (measured by sitting time) ranged from η_p_^2^=0.09 (vigorous days per week) to η_p_^2^=0.58 (sitting; hours per weekday; Table 3). Paired effect sizes (*d*) considering before vs. during confinement were similar between sexes and ranged from *d*=0.32 (female, vigorous physical activities/days/week) to *d*=1.67 (male, sitting/hours per weekday). The greatest difference (*Δd*=0.43) between male (*d*=1.67) and female (*d*=1.24) was observed in sitting/hours per weekday. No significant gender and interaction effects (gender×time) were observed (Table 3).

**Table 3:** Comparison of physical activity parameters and sedentary behavior (sitting time) between males and females before and during confinement.

	Male (*n*=203)			Female (*n*=328)			Variance analysis/Effects *p* (η_p_^2^)		
	Before	During	*d*	Before	During	*d*	Gender	Time	Gender×Time
Vigorous physical activities									
Days/week (d)	2.24±1.22	1.70±1.14	0.46	2.34±1.45	1.90±1.32	0.32	0.124 (0.01)	<0.001 (0.09)	0.478 (0.01)
Minutes/week (min)	37.8±16.4	27.1±15.0	0.68	28.8±16.8	23.1±13.0	0.38	<0.001 (0.06)	**<0.001 (0.17)**	0.002 (0.02)
MET-minutes/week	725±578	417±486	0.58	629±630	400±460	0.42	0.156 (0.01)	**<0.001 (0.15)**	0.166 (0.01)
Moderate physical activities									
Days/week (d)	2.46±1.19	1.75±1.04	0.64	2.39±1.35	1.84±1.26	0.42	0.975 (0.00)	**<0.001 (0.17)**	0.193 (0.01)
Minutes/week (min)	36.1±15.6	26.4±14.3	0.65	32.2±15.6	22.9±12.8	0.66	<0.001 (0.02)	**<0.001 (0.22)**	0.785 (0.00)
MET-minutes/week	388±306	106±57.0	**1.55**	343±285	91.8±51.1	**1.50**	0.033 (0.01)	**<0.001 (0.45)**	0.225 (0.01)
Walking									
Days/walk for at least 10min (d)	4.02±1.73	2.64±1.28	**0.92**	3.50±1.65	2.25±1.24	**0.87**	<0.001 (0.03)	**<0.001 (0.35)**	0.420 (0.01)
Minutes per walking days (min)	37.1±14.1	27.5±14.9	0.66	43.5±18.9	30.9±17.4	0.69	<0.001 (0.03)	**<0.001 (0.22)**	0.100 (0.01)
MET-minutes/week	510±313	267±261	**0.85**	543±372	255±259	**0.91**	0.641 (0.00)	**<0.001 (0.36)**	0.151 (0.01)
Sitting									
Hours per weekday (h)	3.05±1.43	5.64±1.67	**1.67**	2.93±1.35	4.71±1.53	**1.24**	<0.001 (0.05)	**<0.001 (0.58)**	<0.001 (0.05)
All physical activity									
Days/week (d)	2.91±0.95	2.03±0.77	**1.02**	2.74±1.14	1.99±0.89	0.74	0.144 (0.01)	**<0.001 (0.33)**	0.210 (0.01)
Minutes/week (min)	111±33.5	80.9±32.7	**0.91**	105±38.7	77.0±33.1	0.78	0.039 (0.01)	**<0.001 (0.32)**	0.500 (0.01)
MET-minutes/week	1,623±891	790±679	**0.93**	1,515±1,034	747±628	**0.92**	0.206 (0.01)	**<0.001 (0.38)**	0.474 (0.01)

Values are given as mean±SD. Meaningful effects (criteria: *p*<0.05 and η_p_^2^>0.10 and d>0.8) are bolded.

**Figure 1:**
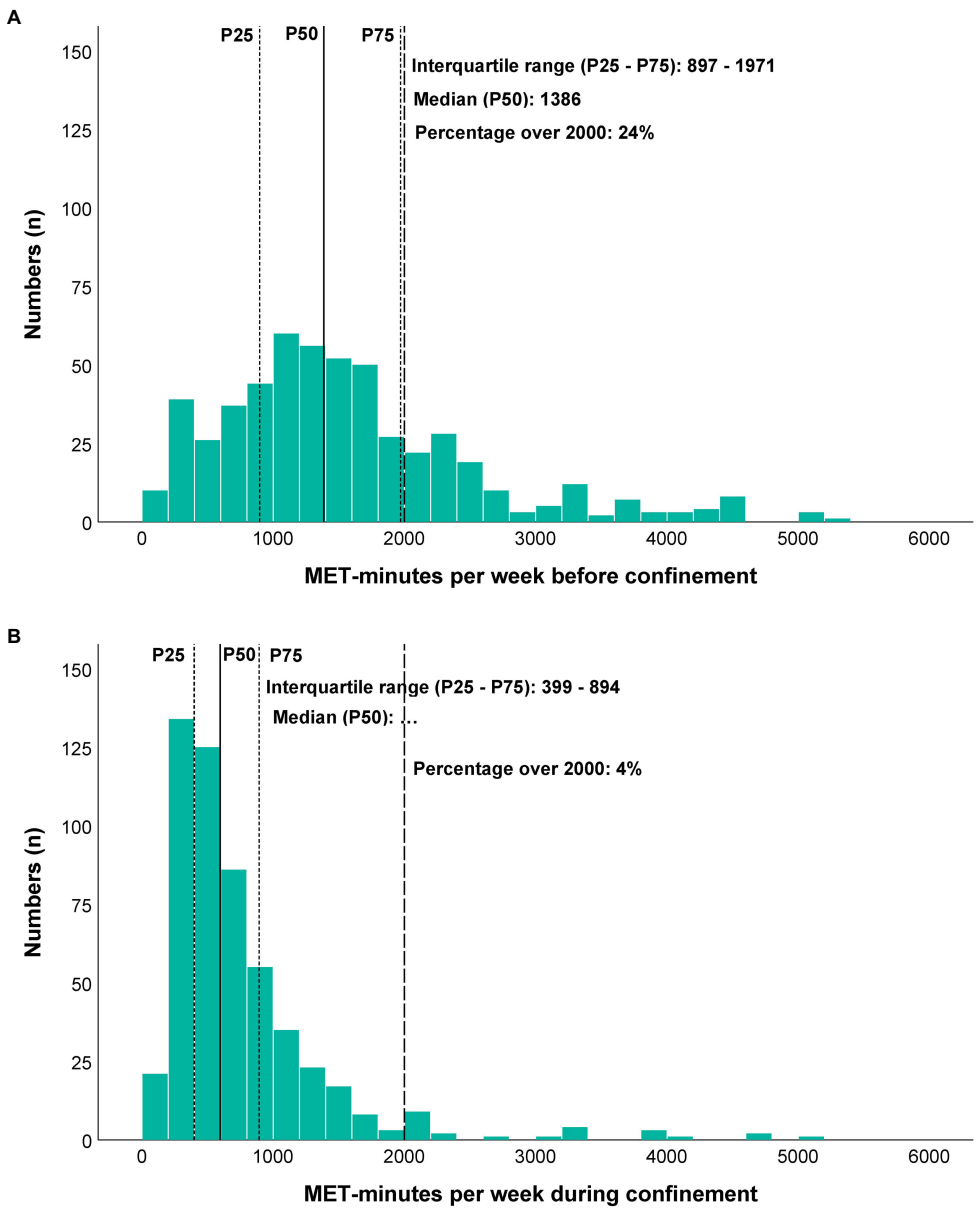
Energy consumption before **(A)** and during **(B)** confinement.

Furthermore, the energy expenditure from PA was decreased in most participants. For example, MET-minutes per week changed from 1,386 (IQR 897–1971; Figure 1A) before confinement to 597 (IQR 399–894; Figure 1B) during confinement. During confinement, only 4% of participants (Figure 1B) were able to perform an energy expenditure >2,000 MET-minutes per week but before confinement, the proportion was 24% (Figure 1A).

### Satisfaction With Life Questionnaire

**Figure 2:**
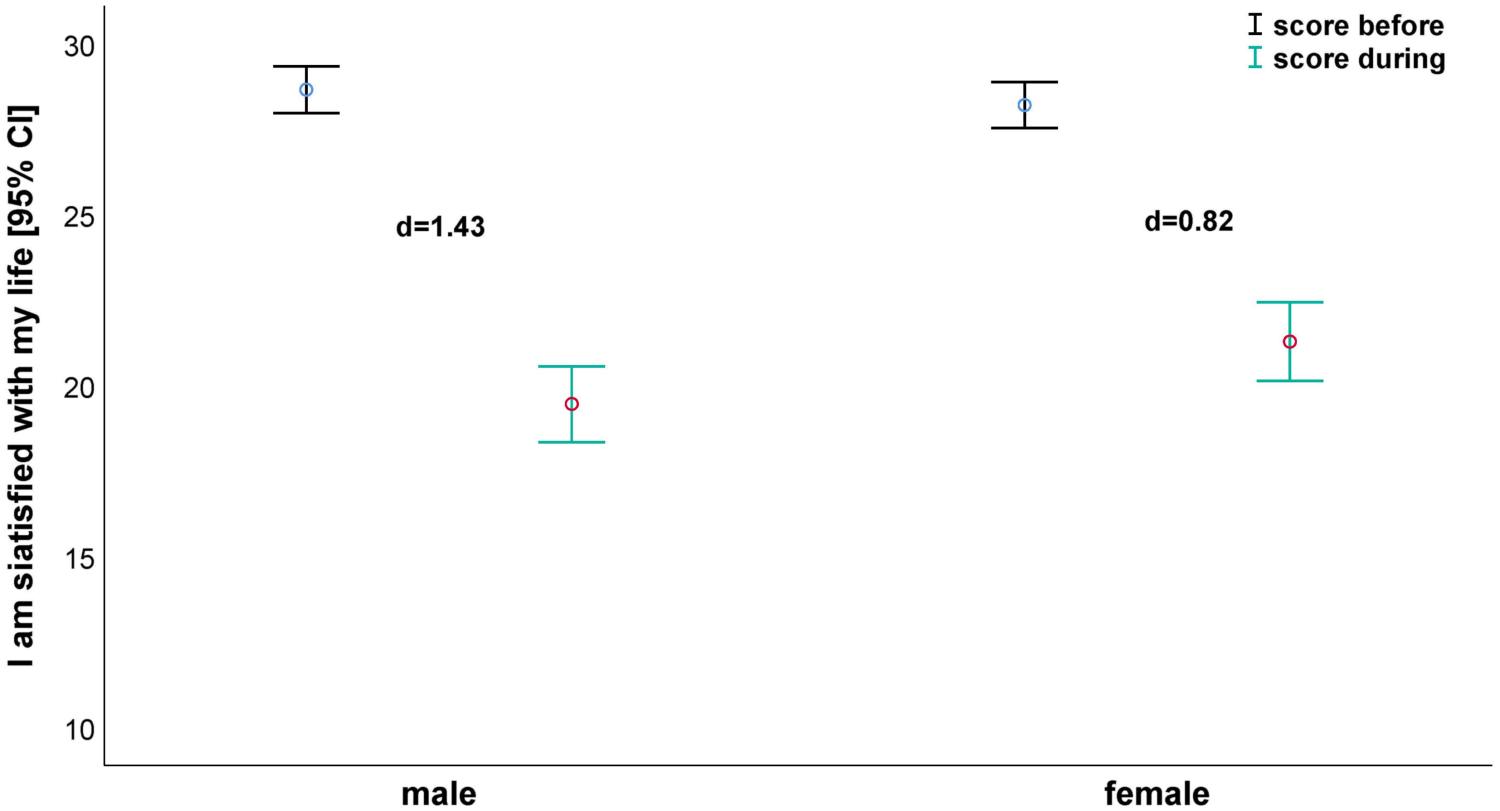
I am satisfied with my life – depending on gender before and during confinement.

Satisfaction with life parameters did not exhibit any relevant gender and interaction (time×gender) effects (Table 4). Time effects reached the *p*<0.05 level in all parameters and ranged from η_p_^2^=0.42 (I am satisfied with my life; Figure 2) to η_p_^2^=0.54 (The conditions of my life are excellent). At the same time, this was the parameter with the largest difference in magnitude between genders (*Δd*=0.98; male: *d*=2.09, female: *d*=1.11).

**Table 4:** Comparison of “satisfaction with life” parameters depending on sex before and during confinement.

	Male (*n*=203)			Female (*n*=328)			Variance analysis/Effects *p* (η_p_^2^)		
	Before	During	*d*	Before	During	*d*	Gender	Time	Gender×Time
In most ways my life is close to my ideal									
Score Q1	27.9±5.74	16.4±6.59	**1.87**	27.6±6.19	19.0±8.32	**1.19**	0.007 (0.01)	**<0.001 (0.49)**	0.001 (0.02)
The conditions of my life are excellent									
Score Q2	30.5±5.19	16.8±7.91	**2.09**	29.4±5.82	20.7±9.93	**1.11**	0.005 (0.02)	**<0.001 (0.54)**	<0.001 (0.05)
I am satisfied with my life									
Score Q3	28.7±4.95	19.5±7.95	**1.43**	28.2±6.18	21.3±10.6	**0.82**	0.231 (0.01)	**<0.001 (0.42)**	0.006 (0.01)
So far I have gotten the important things I want in life									
Score Q4	28.6±5.58	17.7±7.97	**1.52**	27.8±5.79	22.1±8.18	**0.82**	<0.001 (0.03)	**<0.001 (0.46)**	<0.001 (0.08)
If I could live my life over, I would change almost nothing									
Score Q5	27.7±7.74	17.2±6.57	**1.47**	26.1±8.05	18.1±9.30	**0.92**	0.543 (0.01)	**<0.001 (0.43)**	0.001 (0.01)
Total score									
	28.7±4.28	17.5±6.33	**2.11**	27.8±4.65	20.3±7.99	**1.19**	0.017 (0.01)	**<0.001 (0.54)**	<0.001 (0.04)

Values are given as mean±SD. Meaningful effects (criteria: *p*<0.05 and η_p_^2^>0.10 and d>0.8) are bolded. Q, Question.

**Figure 3:**
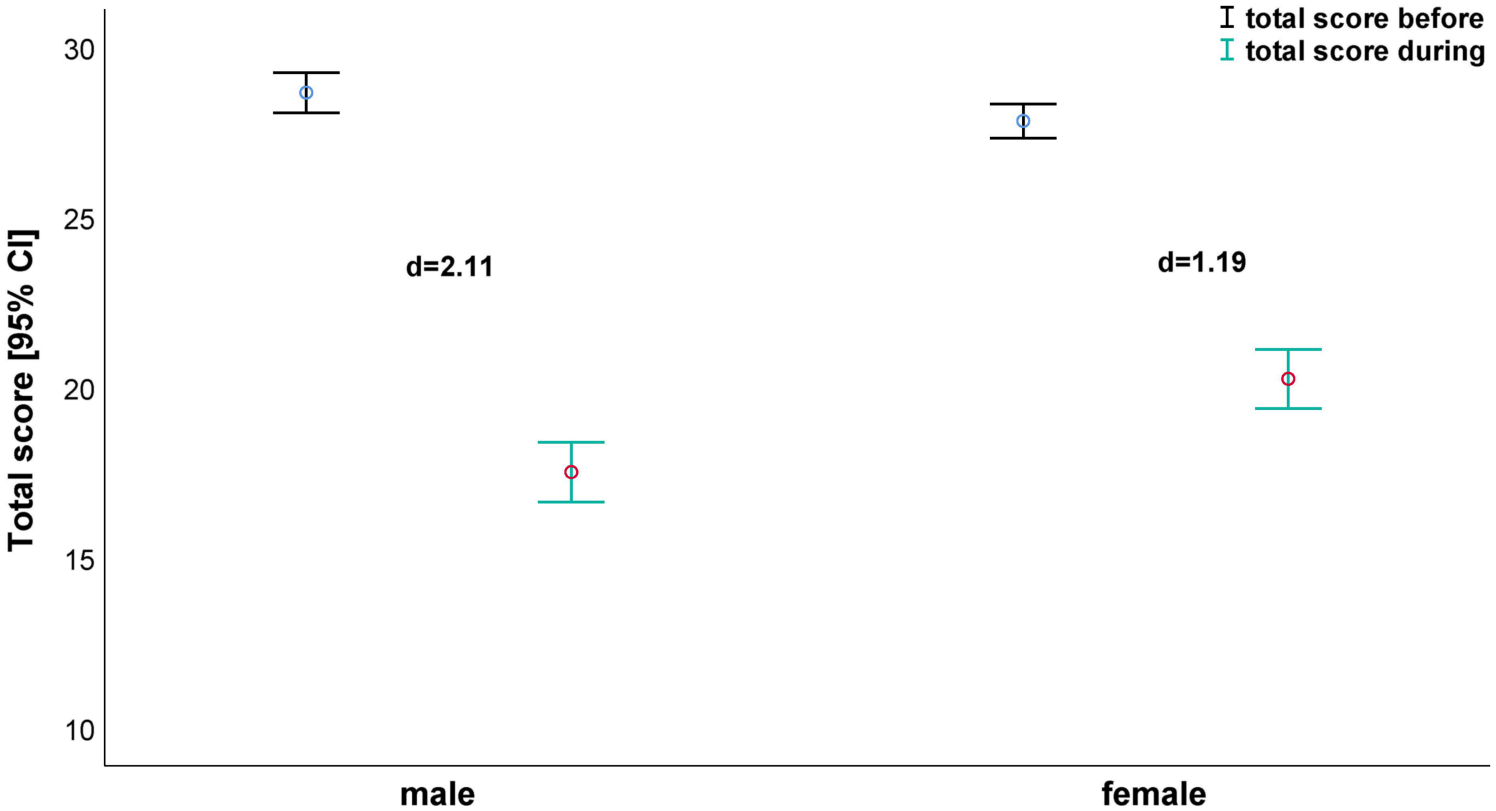
The total score – depending on gender before and during confinement.

The total satisfaction with life (Figure 3) demonstrated the greatest reduction in both males (*d*=2.11; from 28.7±4.28 to 17.5±6.33) and females (*d*=1.19; from 27.8±4.65 to 20.3±7.99).

### Interactions Between Sedentary Time, PA, and Satisfaction With Life Questionnaire

**Figure 4:**
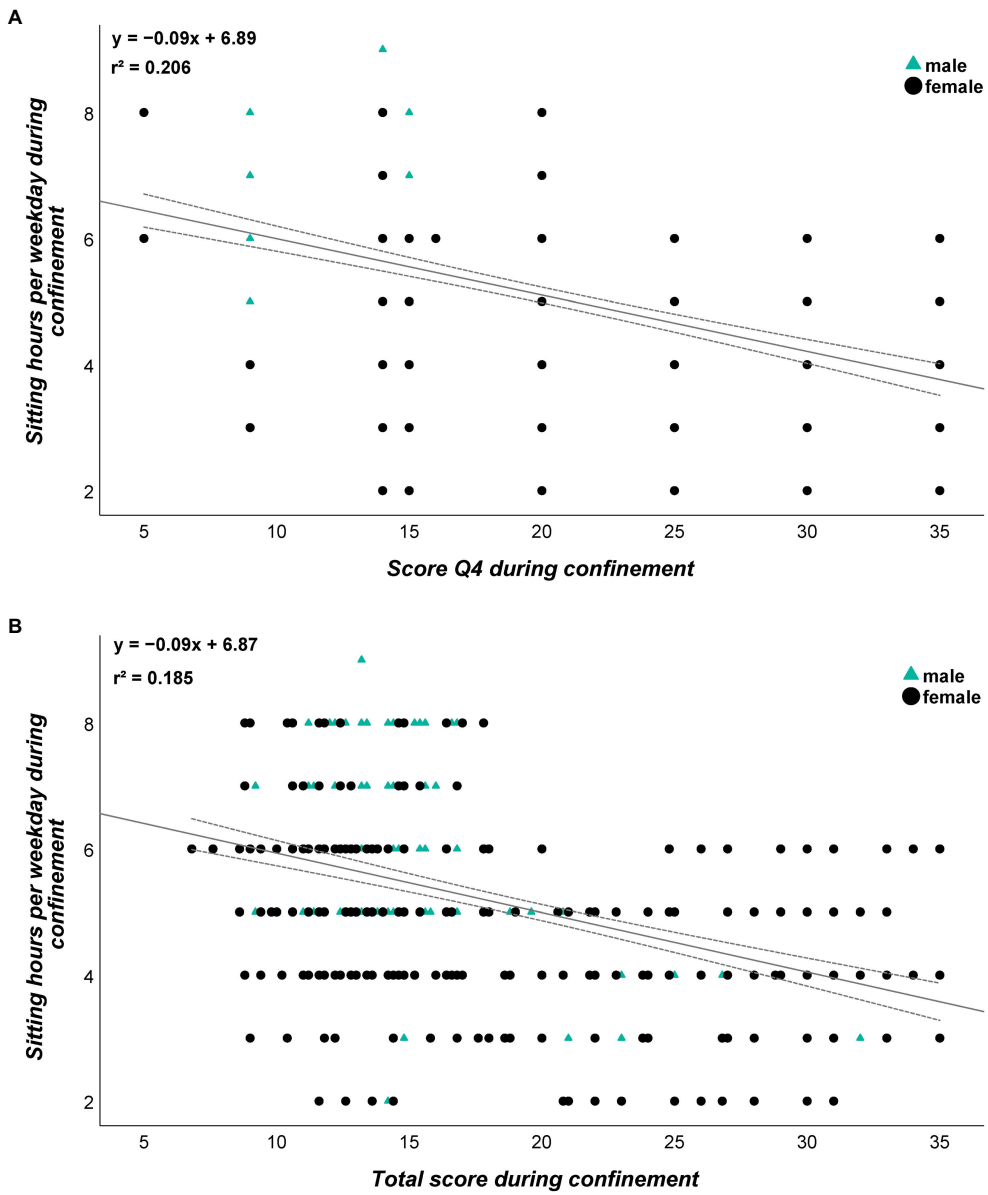
Relationship between sitting time and Q4 **(A)** and sitting time and total score **(B)** depending on gender. Please note that one dot can represent several subjects.

For a more detailed analysis of the dependences between the used PA and satisfaction with life parameters, we calculated bivariate correlations between these parameters before and during confinement. No relevant (*r*>0.5) correlations between PA and SL statements were observed at either time point. The largest correlations were detected for sitting time during confinement and Q4 score (Figure 4A) and total score of SL (Figure 4B).

## Discussion

The COVID-19 pandemic has caused many citizens to be asked to stay at home, associated with reduced PA and increased mental health burden (Lee et al., 2012; Hemphill et al., 2020). This study aimed to evaluate effects of home confinement on PA and SL of university students in Qatar. Home confinement was associated with a significant decrease in walking per week and an increase in sitting time. Moreover, PA at intense and moderate levels of intensity declined by home confinement. Total score for SL also decreased by ~32% (male: 39%; female: 27%). These results suggest that there may be a significant risk of detrimental psychosocial strain during home confinement because of the pandemic. Furthermore, this study proposes that the inability to adapt has a direct effect on university students’ PA participation.

### Impact of COVID-19 on Physical Activity

Previous research has established the positive health benefits of PA. Evidence is in the direction of a positive relationship between non-communicable diseases risk and physical inactivity (Tremblay et al., 2011; Hermassi et al., 2021a). To these ends, many governmental agencies have developed PA guidelines not only as a preventive strategy for chronic diseases, but also for psychological benefits (Lee et al., 2012; King et al., 2019). Recent multicenter studies showed that COVID-19 home confinement increased the percentage of physically inactive individuals (+15%; Ammar et al., 2020a). This is supported by our study findings that highlight the falling trend in walking time. Previous studies completed during lockdown similarly report PA reductions during confinement and increased sedentary time as a result of poor weather conditions serving as a barrier for going outdoors (Hermassi et al., 2021a).

In this study, the number of walking days per week for at least 10min was decreased in university students 35% (female: 36%; male: 34%). Concomitantly, time per walk decreased 28% (female: 29%; male: 26%). Therefore, energy expenditure of walking per week decreased from 543±372 MET-minute/week to 255±259 MET-minute/week for female and in men from 510±313 MET-minute/week to 267±261 MET-minute/week. Walking is a component of daily life, so decreased walking time in our university students therefore reflects a sedentary lifestyle. An international online survey published in April 2020 reported that days/week of walking decreased 35% during home confinement (Ammar et al., 2020a). Moreover, this study reported that minutes/day of walking decreased 34% and MET values of walking were 43% lower during home confinement.

Hermassi et al. (2021a) reported a decrease in frequency of high intensity PA during home confinement from 40 to 23% in women and from 32 to 30% in men, in the general population in Qatar. In addition, the greatest difference between male (*d*=1.97) and female (*d*=1.30) was observed in moderate PA (MET-minute/week). Significant gender effects were observed for 46% (6/13) of the investigated parameters. However, Hermassi et al. (2021a) concerned the general population in Qatar, whereas this study focused specifically on university students which may explicate the divergent result and give reason no interaction effects (gender×time) were observed, as university students may have more homogenous lifestyles between genders at this stage of life.

Giustino et al. (2020) reported energy expenditures at vigorous intensity decreased from 520±372 MET-minute/week to 238±205 MET-minute/week in women and from 663±320 MET-minute/week to 323±187 MET-minute/week in men by home confinement. However, Ammar et al. (2020a) indicate that the number of days/week and minutes/day of vigorous intensity PA during confinement decreased 23 and 33%, respectively. Moreover, vigorous intensity METs were 37% lower compared to before during home confinement. Contrarily, in a survey measuring PA during lockdown, intensive PA levels among Canadian highly active adults [Moderate to Vigorous Physical Activity (MVPA) of 302±186min per week] did not decrease (Lesser and Nienhuis, 2020). Their intensive PA consisted of outdoor exercise in form of running, cycling, and walking. However, the reported amount of intensive and moderate PA in this study decreased. The differences in the results of these studies could be due to the differences between individual’s reason behind PA involvement such as enjoyment or health benefits associated with this type of activity.

According to the Eurobarometer, 34% of the Spanish adult population do not achieve the minimum recommended PA, and 36% spend most of the day seated (national health surveys in Spain). Conversely, Rogowska et al. (2020) suggested 43% of university students were physically active during the coronavirus lockdown (i.e., ≥150min/week of PA). Likewise, 56% of Chinese college students were physically active at moderate or vigorous levels during the national quarantine (Zhang et al., 2020). This lead Rogowska et al. (2020) to suggest the sample of Ukrainian students may be less involved in PA than Chinese undergraduates. Furthermore, the number of active students decreased significantly in comparison with the situation before the COVID-19 outbreak in Ukraine, which is consistent with some previous studies (Nguyen et al., 2020; Wang and Zhao, 2020). That being said, Zhang et al. (2020) assessed weekly PA during the last 2weeks according to three categories: light, moderate, and vigorous using the full-length IPAQ. Thus, these studies are not fully comparable with the present investigation as we utilized the IPAQ-SF.

In our population, our inactive population (i.e., those not meeting PA guidelines) before confinement was lower (25% corresponding to 75min/week vigorous PA) than that of the Eurobarometer. This may be explained by the fact that university students recruited for the study were more active as evidenced by the number of participants undertaking >225min/week of vigorous activities, which could be attributed to the fact that the questionnaire was shared by institutions linked to sport and exercise.

Sitting and sedentary time increased considerably in this study, most likely due to the alteration in daily activities (walking, cycling, or transport to work, etc.) and the prolonged stay at home. Young people and students spent more time seated during confinement, possibly due to the enforced e-learning environment, which encourages sedentary behavior and, lots of screen-based activities time (King et al., 2019). Likewise, according to the socioecological model (Lesser and Nienhuis, 2020), in a comparable framework where social or environmental barriers promote an inactive lifestyle (loneliness, social isolation, and when the season changes), more sedentary behavior and less time being spent on light, moderate and vigorous PA have been reported (Ammar et al., 2020b; Hermassi et al., 2021a). Hence, an involuntary prolonged stay at home may encourage sedentary behavior as well as during confinement caused by COVID-19.

Qi et al. (2020) demonstrated that almost 60% of Chinese adults had greater sedentary time during the COVID-19 pandemic (i.e., 4h longer per day). In terms of sedentary behavior in this study, sitting time increased more in men who also significantly reduced moderate intensity activities, while female students increased these activities. Our data showed a high percentage of individuals with depression and anxiety symptoms (anxiety or depression: 13%). Such results were expected as the current pandemic is a time of uncertainty and concern for many people. This can affect individuals on a physical level, but also on a psychological level. Indeed, many people will experience reactions of symptoms of stress, anxiety, and depression.

### Impact COVID-19 on Satisfaction With Life

QoL is related to life satisfaction refers to the subjective evaluation of current life quality, which is an essential indicator of psychological health and well-being. Rogowska et al. (2020) reported that approximately one-third of the university students suffered from various forms of depression and anxiety (from moderate to severe symptoms).

The fear of being infected by COVID-19 along with stress produced by restrictive isolation measures has been negatively related to people’s SL (Ammar et al., 2020a,c; Hermassi et al., 2021b). Results from this study support those of Hermassi et al. (2021a), who demonstrated that factors such as economic loss, flawed information from Public Health Authorities, and long periods of quarantine were some of the stressors during home confinement and main cause of reduction in SL (Alajmi et al., 2020). Conversely, Li et al. (2020) claimed the relationship between life satisfaction and self-compassion in the Chinese population during the COVID-19 quarantine did not differ between the sexes. For example, women with high levels of positive coping did not demonstrate higher life satisfaction compared to men. These findings support the present study that suggests male university have a preservation of SL during pandemic isolation periods.

The absence of clear guidelines and confusion about the purpose of quarantine has adverse influences on mental health and life satisfaction (Al Kuwari et al., 2020; Alajmi et al., 2020) since in situations of existential uncertainty, people redouble efforts to preserve a shared and coherent vision of social reality (Li et al., 2020). Consequently, a reliable comparison of mental health during the COVID-19 pandemic may be difficult to achieve. A cross-sectional study in Vietnam by Nguyen et al. (2020) demonstrated health literacy was a protective factor for depression and life satisfaction during the current pandemic. These findings reaffirm those results obtained in this study, where the perception of sufficient information received has proven to be one of the main contributing factors to greater life satisfaction during forced home confinement.

The present findings revealed a significant (*p*<0.001) but not meaningful (η_p_^2^=0.08) interaction effect for the statement “So far I have gotten the important things I want in life” as the reduction was less in females (24%) than males (38%). The time effect reached *p*<0.001 in all satisfaction with life parameters. Ammar et al. (2020a) showed SL decreased 16% during home confinement. This overall score is a factor of three questions (Q1–Q3), and the decrease from “before” to “during” confinement ranged from 27% (Q3) to 35% (Q1, Q2). These negative effects have also been reported in a recent COVID-19 series highlighting that people under quarantine conditions report more symptoms of psychological distress. Furthermore, some symptoms appeared to persist after quarantine has ceased (Giovanni et al., 2004). In China, COVID-19’s resultant social distancing reduced life satisfaction and increased distress (Kopietz et al., 2010). The present findings corroborate these previous reports, substantiating the risk of psychosocial strain during home confinement periods.

A study carried out in Brazilian and Portuguese populations suggested sociodemographic factors such as higher educational level, living with family members or a partner in the period of social isolation, or low levels of depression, being female, and being a student were significantly linked with heightened levels of life satisfaction (Torales et al., 2020). However, Giovanni et al. (2004) suggested factors, such as marital status, having children, educational level, and age were not associated with increased levels of life satisfaction.

Ammar et al. (2020c) indicated that COVID-19 home confinement had a negative effect on mental health, mood, and feelings. In terms of prevalence, more individuals (+13%) reported lower mental wellbeing during, compared to before, home confinement. Mood and feelings responses showed a 45% increase in depressive symptoms, with more people (+10%) showing depressive symptoms during, compared to before, home confinement. The ECLB-COVID-19 survey revealed an increased psychosocial strain triggered by home confinement. To mitigate this risk of poor mental health and to foster an active and healthy confinement lifestyle (AHCL), a crisis-oriented interdisciplinary intervention is urgently needed.

It seems university students experience consistently higher rates of mental health problem than other populations, which may be elevated by the COVID-19 pandemic and lockdown restrictions. The findings from the correlation analysis suggest exposure to COVID-19, and individual differences in the perception of the coronavirus impact showed significant but weak associations with SQL, so other factors may be more important in explaining mental health among Qatar university students.

### Strengths, Limitations, and Perspective

This study presents some limitations. Firstly, the online method of recruitment has its limitations. Data collection took place during COVID-19 lockdown, students’ lectures and seminars were online, and students utilized web-based technologies. Second, because of the limited resources available and the quarantine, the snowball sampling strategy and online self-report survey were adopted, which might be subject to participation bias, social desirability bias, and shared method variance.

In addition, although the questionnaire collected data about the previous 7days, in our study, we requested information beyond a week, which may be justified due to the unprecedented situation and the lack of time available to manage more appropriate study design and methods. The questionnaire had to be done during pandemic and then during different time points in pandemic period. This should be taken into account when interpreting the results.

The strength of this study was the rapid data generation during the pandemic using a widely distributed anonymous cross-disciplinary survey pro-vided in two languages. Further research may include a more balanced proportion of the sexes in the study sample. Moreover, this study’s results cannot be generalized into other adult populations, since participants in this study were exclusively university students. Some demographic variables were not included in the questionnaire; among a few were ethnicity, income, employment, marital status, or household members. There were some methodological issues and possible limitations (a) cross-sectional design that assessed “before confinement,” and (b) disuse of cookie-based or I.P.-based duplicate protection to exclude duplicates. However, we chose to avoid I.P. or cookie safety measures, since more than one family member may use the same computer and therefore the same I.P. address.

Given that the home confinement was a sudden measure in most countries, we were unable to develop and disseminate the survey “before” home confinement, to avoid recall bias. Use of previous datasets may overcome this limitation, but this would have required big data handling and advanced computer science skills which fall outside the scope of this study (Kopietz et al., 2010). Additionally, confounding factors not accounted in this present study due to need for rapid data gathering, such as the presence of children in the house, job loss, losing friends or relatives, divergent professions, various types of PA might have had an effect on SL, and experienced stress. These factors could be considered for future studies.

## Conclusion

In this study, we observed PA and SL decreased in university students in Qatar during the COVID-19 pandemic. Participants spent more time inactive and more time sitting. Besides the stressor associated with the illness itself, survey results affirmed a destructive effect of home confinement on PA, increased sitting time, and sedentary behavior. We propose the introduction of online PA training on e-learning platforms (e.g., Moodle, MS Teams, and Zoom), conducted by a professional sports coach in the field of aerobic and anaerobic exercises varying in intensity level (light, moderate, and vigorous), to be chosen by students depending on their movement abilities and interests. For university students with severe symptoms of anxiety and depression, supportive interventions can include physical exercises performed in conjunction with individual psychological online therapy. The observed reduction in physical activity and energy expenditure is very likely associated with an increase in body weight. Obesity, in turn, is an important risk factor for diabetes, hypertension, and cardiovascular diseases. The resulting reduction in quality of life and life expectancy should in future be taken into account when coping with pandemics in order to avoid irreparable collateral damages (proportionality of measures).

## Data Availability Statement

The raw data supporting the conclusions of this article will be made available by the authors, without undue reservation.

## Ethics Statement

The studies involving human participants were reviewed and approved by The Qatar University’s Institutional Review Board (QU-IRB 1350-EA/20). The patients/participants provided their written informed consent to participate in this study.

## Author Contributions

SH and RS: conceptualization. SH: methodology, software, investigation, writing – original draft preparation, visualization, project administration, and funding acquisition. SH and AS: validation. EB, EA, LA-K, NA, NM, and AA: formal analysis. RS: data curation and supervision. AS and LH: writing – review and editing. All authors contributed to the article and approved the submitted version.

## Funding

The research leading to these results has received funding from Qatar University Student Grant QUST-1-CAS-2021-6. The funding achieved herein are solely the responsibility of the authors.

## Conflict of Interest

The authors declare that the research was conducted in the absence of any commercial or financial relationships that could be construed as a potential conflict of interest.

## Publisher’s Note

All claims expressed in this article are solely those of the authors and do not necessarily represent those of their affiliated organizations, or those of the publisher, the editors and the reviewers. Any product that may be evaluated in this article, or claim that may be made by its manufacturer, is not guaranteed or endorsed by the publisher.

## Footnotes

^1^ https://policies.google.com/privacy?hl=en
